# Resting-state brain connectivity correlates of musical sophistication

**DOI:** 10.3389/fnhum.2023.1195996

**Published:** 2023-09-29

**Authors:** Anja-Xiaoxing Cui, Sarah N. Kraeutner, Negin Motamed Yeganeh, Nancy Hermiston, Janet F. Werker, Lara A. Boyd

**Affiliations:** ^1^Department of Musicology, University of Vienna, Vienna, Austria; ^2^Department of Psychology, University of British Columbia, Vancouver, BC, Canada; ^3^Department of Psychology, University of British Columbia, Kelowna, BC, Canada; ^4^School of Music, University of British Columbia, Vancouver, BC, Canada; ^5^Brain Behaviour Lab, Department of Physical Therapy, University of British Columbia, Vancouver, BC, Canada

**Keywords:** resting-state fMRI, musical sophistication, music training, functional connectivity, music perception, music production, music imagery

## Abstract

**Introduction:**

A growing body of research has investigated how performing arts training, and more specifically, music training, impacts the brain. Recent meta-analytic work has identified multiple brain areas where activity varies as a function of levels of musical expertise gained through music training. However, research has also shown that musical sophistication may be high even without music training. Thus, we aim to extend previous work by investigating whether the functional connectivity of these areas relates to interindividual differences in musical sophistication, and to characterize differences in connectivity attributed to performing arts training.

**Methods:**

We analyzed resting-state functional magnetic resonance imaging from *n* = 74 participants, of whom 37 received performing arts training, that is, including a musical instrument, singing, and/or acting, at university level. We used a validated, continuous measure of musical sophistication to further characterize our sample. Following standard pre-processing, fifteen brain areas were identified *a priori* based on meta-analytic work and used as seeds in separate seed-to-voxel analyses to examine the effect of musical sophistication across the sample, and between-group analyses to examine the effects of performing arts training.

**Results:**

Connectivity of bilateral superior temporal gyrus, bilateral precentral gyrus and cerebellum, and bilateral putamen, left insula, and left thalamus varied with different aspects of musical sophistication. By including these measures of these aspects as covariates in *post hoc* analyses, we found that connectivity of the right superior temporal gyrus and left precentral gyrus relate to effects of performing arts training beyond effects of individual musical sophistication.

**Discussion:**

Our results highlight the potential role of sensory areas in active engagement with music, the potential role of motor areas in emotion processing, and the potential role of connectivity between putamen and lingual gyrus in general musical sophistication.

## 1. Introduction

A growing body of research shows that training on a musical instrument may impact brain structure and function through its frequent engagement of sensory, motor, and imagery areas. For example, a recent meta-analysis of 58 studies using the anatomic/activation likelihood estimation method suggested that there are structural and functional differences between levels of musical expertise in sensory, motor, and interoceptive brain areas (Criscuolo et al., 2022). These areas are also implicated in a similar meta-analytic work investigating different types of music processing tasks, including tasks of music perception, music performance, and music imagery (Pando-Naude et al., 2021).

However, these differences may not be specific to music training but due to training in the performing arts in general. Performing arts include a variety of forms but share the experience of being overtly performed, mostly in front of an audience on a stage. A recent study of the insular connectivity assessed with resting state functional magnetic resonance imaging (rs-fMRI) revealed significant differences between performing artists and a control group of participants, but not between the musicians and dancers (Gujing et al., 2019). Similarly, there are reports of structural changes and functional changes in sensory, motor, and interoceptive areas after dance training (Karpati et al., 2015; Rehfeld et al., 2018), between participants and non-participants in a theater acting intervention (Rajesh et al., 2021), and between traditional Chinese opera actors and control participants (Zhang et al., 2018).

Because past work largely used categorical distinctions of musical expertise, we know little about how patterns of activity relate to interindividual differences in musical sophistication. Musical sophistication is a construct that includes a wide range of musical expertise, e.g., singing abilities, and is not exclusive to those who have received formal training (Müllensiefen et al., 2014). Moreover, task fMRI studies of gradient musical sophistication are hard to realize given the difficulty of finding musical tasks with graded difficulty. Instead, rs-fMRI may offer insights on how individual levels of musical expertise relate to brain activity.

Resting state-fMRI activity is thought to reflect the history of co-activation of functionally connected regions (Guerra-Carrillo et al., 2014). Differences in co-activation illustrated by rs-fMRI between experts and non-experts may be reflective of the activities in which the experts under study have expertise. These differences can be both positive and negative. Co-activation during rest may be greater for experts, interpreted as better connectivity in experts, or greater for non-experts, interpreted as better efficiency and selectivity in experts through automatization of tasks.

Indeed, both patterns are reported in past work. Some studies have revealed increased rs-fMRI activity in the insula of “expert” musicians—often defined as students majoring in music—(Luo et al., 2014; Tanaka and Kirino, 2016b), a brain area thought to serve as a link between sensory, motor, and interoceptive areas (Kurth et al., 2010). Other work suggests that musicians show decreased brain activity and thus more selective activation of the striatal network (Tanaka and Kirino, 2016a). Similarly, past comparisons of experts and non-experts in motor domains have identified functional modifications of areas involved in motor execution and attentional control shown both as increases and decreases of rs-fMRI activity (Cantou et al., 2018).

Our aim is to extend this research and clarify how rs-fMRI activity relates to interindividual levels of musical sophistication in young adults. Musical sophistication as conceptualized for the Goldsmiths Musical Sophistication Index (Gold-MSI) (Müllensiefen et al., 2014) contains five aspects: Active engagement, perceptual abilities, musical training, singing abilities, and emotions. Active engagement assesses how much time and money a respondent invests in music and music-related activities. Perceptual abilities and singing abilities refer to how easily a respondent can perceive music, e.g., hear mistakes or differences between performances, and how well a respondent can sing. Some respondents are highly attuned to the emotions conveyed by music; this is conceptualized as the emotions aspect of musical sophistication. Musical training is measured by asking respondents to estimate past training on instruments and in music theory, as well as time spent practicing. General musical sophistication encompasses all aspects.

To assess how these interindividual levels of musical sophistication relate to the connectivity of sensory, motor, and interoceptive areas, we selected 15 regions of interest (ROI) based on recent meta-analytic work (Pando-Naude et al., 2021; Criscuolo et al., 2022). These ROIs were identified in this meta-analytic work as the core region of eight clusters related to performance in music perceptual tasks, five clusters related to performance in music production tasks, and four clusters related to performance in music imagery related tasks. The ROIs are listed in Table 1.

**Table 1:** Seeds included in seed-voxel analyses, organized by process.

	Seed region
Sensory	L Superior temporal gyrus (anterior)
	R Superior temporal gyrus (anterior)
	L Superior temporal gyrus (posterior)
	R Superior temporal gyrus (posterior)
	L Heschl’s gyrus
	R Heschl’s gyrus
Motor	L Precentral gyrus
	R Precentral gyrus
	L Superior parietal
	L Middle frontal gyrus
	L Cerebellum (lobule III)
Interoceptive	L Putamen
	R Putamen
	L Insula
	L Thalamus

The ROIs include the bilateral auditory cortices. A large number of studies have concluded that the activity and structure of the bilateral auditory cortices relates to performance in music perceptual tasks, for example, in pitch perception (Schneider et al., 2002), the differentiation between different modes of music (Foo et al., 2016), or melodic contour perception (Taddeo et al., 2022). Thus, we hypothesized that the connectivity of these areas would relate to the music perceptual abilities aspect of musical sophistication. We further hypothesized that the connectivity of the included motor areas would relate to the music training aspect of musical sophistication, given the body of literature demonstrating the effects of music training on such areas (e.g., Kleber et al., 2016, 2017).

The bilateral putamen, the left insula, and the left thalamus, grouped in Table 1 under interoceptive areas, have been implicated in a number of different studies: Their activity is known to be modulated by music and performing arts training for example (Luo et al., 2014; Kleber et al., 2017; Zamorano et al., 2017; Gujing et al., 2019; Ai et al., 2022), making relationships to the training aspect of musical sophistication as well as differences between participants with a performing arts background and those without likely. Most recently for example, insular connectivity was related to singing training (Zamorano et al., 2023).

Not all performing arts require the production of music, yet all do involve the perception of auditory stimuli and the movement of one’s body, i.e., musicians move their bodies to produce music, dancers move their bodies to music, and actors move their bodies while delivering speech. Thus, connectivity differences of sensory and motor areas are also likely between participants with a performing arts background and those without.

In summary, we hypothesized that the connectivity of sensory, motor, and interoceptive brain areas relates to interindividual levels of differing aspects of musical sophistication. We further explored whether or not the connectivity of these areas is modulated through formal performing arts training by studying students receiving formal performing arts training and those who are not. As we expected that participants who receive performing arts training to have higher musical sophistication, we controlled for this factor when investigating group differences in connectivity.

## 2. Methods

### 2.1. Participants

We recruited 76 participants, of whom 38 were receiving performing arts training, through enrolment in instrumental music, singing, and/or acting classes at university level. The other participants were recruited from language classes offered at the same university and from a group of varsity runners. These classes were selected so as to ensure participants were also engaging regularly with structured auditory input and/or motor sequences as part of their university experience. Participants were excluded if they were younger than 18 or older than 40, had a major psychiatric diagnosis, neurological disease or damage or contraindications to MRI.

One participant was excluded from analysis due to an incidental finding on MRI scanning and another participant was excluded from analysis due to excessive head motion in the scanner. Thus, we analyzed data from *n* = 74 participants, of whom 37 were receiving performing arts training. Of our participants included in the analyses, 42 self-identified as female, and 32 as male. The average age of our participants was *M* = 20.84 years, *SD* = 2.98 years. On average, participants who were receiving performing arts training reported *M* = 10.69 years, *SD* = 5.05 years of past formal music training. Participants who were not receiving performing arts training reported *M* = 4.29 years, *SD* = 4.11 years of past formal music training. Of the participants included in the analyses, 15 were enrolled in music classes but no acting classes, 17 were enrolled in music and acting classes, and 5 were enrolled in acting but no music classes.

### 2.2. Data collection

Participants provided self-assessments of their musical sophistication using the Gold-MSI (Müllensiefen et al., 2014). The Gold-MSI provides a continuous measure of general musical sophistication as well as continuous measures of five different aspects of musical sophistication: active engagement, perceptual abilities, musical training, singing abilities, and emotions.

MRI data were obtained in a 3 T Philips Elition MRI scanner. We collected a high-resolution T_1_ scan from each participant (TR = 7.4 ms, TE = 3.7 ms, flip angle θ = 6°, FOV = 256 mm, 160 slices, 1 mm thickness, scan time = 3.2 min). Rs-fMRI was recorded with a single shot EPI sequence (TR = 2,000 ms, TE = 30 ms, flip angle θ = 90°, voxel dimension = 3 mm^3^ with 1 mm gap, 36 slices, FOV = 240 mm × 240 mm, scan time = 8.2 min). During the resting state functional MRI scan, participants were instructed to keep their eyes on a fixation cross and to stay awake.

### 2.3. Data analysis

Gold-MSI data were processed using the templates provided by the instrument’s authors (Müllensiefen et al., 2014). Reverse-coded items are rescored before individual item answer scores are added together for each subscale.

All f/MRI data were processed using the CONN toolbox (CONN v.17) (Whitfield-Gabrieli and Nieto-Castanon, 2012). Standard preprocessing (including motion correction, co-registration, and resampling) was conducted using SPM12 functions integrated in the CONN toolbox (Penny et al., 2007). Specifically, functional data were corrected for motion using the SPM12 realign and unwarp procedure with default settings, with any scans that exceeded 5.0 mm mean framewise displacement excluded from further analyses.

Functional images were co-registered to their corresponding T1-weighted structural images. Using SPM12 unified segmentation and normalization procedures, structural images were normalized to SPM’s Montreal Neurological Institute 2-mm T1 template by an affine transformation and non-linear registration, with the same estimated non-linear transformation then applied to the functional data. Functional and anatomical data were then resampled into 2 and 1 mm isotropic voxels, respectively, and functional data was spatially smoothed with a Gaussian kernel (FWHM = 6 mm).

To remove the effects of nuisance covariates, noise components were then regressed out of the fMRI data, along with white matter signal, and cerebrospinal fluid signal at the individual-level (Whitfield-Gabrieli and Nieto-Castanon, 2012). The temporal signals in the 4-dimensional volume were linearly detrended and band-pass filtered (0.01–0.08 Hz) to remove undesired components.

In line with our research objectives, 15 ROIs were identified *a priori* based on recent meta-analytic work (Pando-Naude et al., 2021; Criscuolo et al., 2022) which assessed brain activity subserving music processing. In particular, ROIs were related to music perception, music production, and music imagery, encompassing frontal, posterior parietal, and temporal regions, as well as medial regions (e.g., localized to the cingulate, thalamus). A complete list of ROIs is presented in Table 1.

Seed-to-voxel connectivity maps were generated across the whole brain at the participant level, using our ROIs. Functional networks generated from each individual seed were investigated separately (i.e., resulting in 15 separate analyses, and not averaged over seed regions within each network). The mean BOLD time course from each seed was extracted, with correlation coefficients calculated with the time course of each voxel throughout the whole brain, with resultant correlation maps generated. The resulting coefficients obtained seed-to-voxel analyses were Fisher z-transformed and extracted for the purposes of group-level analyses and brain-to-musical sophistication correlation analyses. All second-level analyses were performed in CONN and used a significance threshold of *p* < 0.05 (two-tailed), FDR-corrected for multiple comparisons.

To determine how rs-fMRI is modulated by musical sophistication, separate regressions were conducted on resultant connectivity with Gold-MSI scores entered as predictor variables. To determine how rs-fMRI is modulated by performance arts training, between-group comparisons for those who received formal performing arts training (*n* = 37) vs. those who did not (*n* = 37) were conducted on the participant-level correlation matrices.

In instances where between-group effects resulted for seeds showing an association between Gold-MSI scales and rs-fMRI, *post hoc* comparisons were conducted to determine if such a group effect could be explained by differences in musical sophistication. Specifically, between-group comparisons were conducted on the participant-level correlation matrices with the relevant Gold-MSI scale scores included as a covariate so as to account for differences musical sophistication across participants.

To summarize, we assessed (1) correlations of connectivity and musical sophistication, (2) effects of performing arts training and whether these effects may be explained by variations in musical sophistication. While it is possible that increased Type 1 error will arise given the number of seeds included in our analyses and large numbers of statistical (i.e., multiple) comparisons performed, all of our results are corrected for multiple comparisons within each seed using the fairly conservative method of family-wise error (FWE). However, acknowledging this possibility, we believe this “exploratory” work is valuable for the field given that our study includes a large sample size relative to the field, and limited work has been conducted employing measures of functional connectivity to further our understanding of how performing arts training impacts the brain.

## 3. Results

### 3.1. Musical sophistication

**Figure 1:**
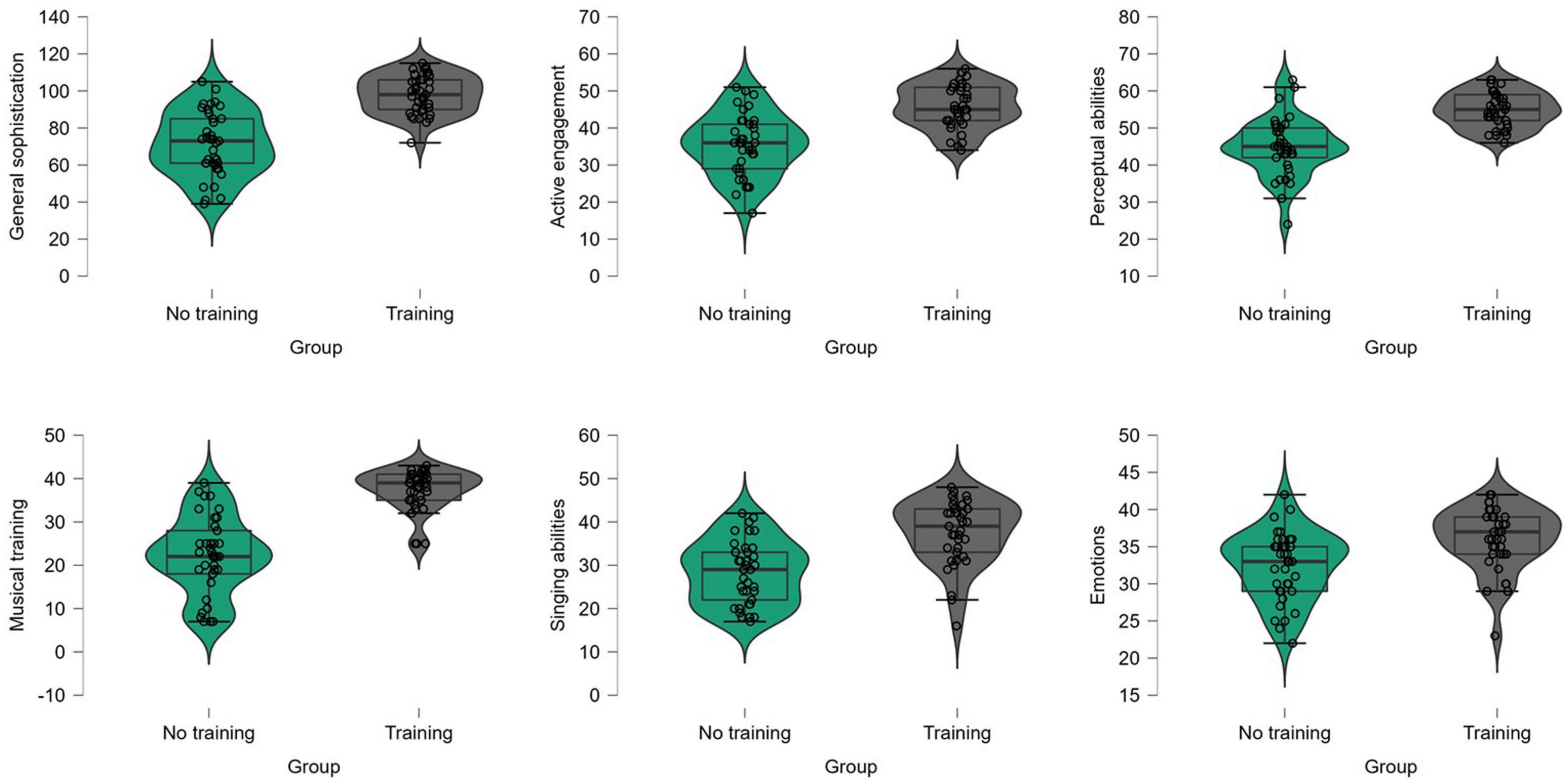
Violin plots of Gold-MSI subscales overlaid with boxplots. Data of participants in the performing arts training group are shown in grey while data of participants in the no performing arts training group are shown in green.

Average Gold-MSI scores for each subscale are reported in Table 2 along their corresponding percentiles in the norming sample reported by the instrument’ authors (Müllensiefen et al., 2014). Violin plots are shown in Figure 1 overlaid with boxplots. We performed independent samples *t*-tests to investigate differences between groups. While participants in the performing arts group had higher Gold-MSI scores, *p*s < 0.001, many participants in the no performing arts training also had high Gold-MSI scores. We report the results of the independent samples *t*-tests in Table 2.

**Table 2:** Average Gold-MSI scores, corresponding percentiles, and results from independent samples *t*-tests.

	Performing arts training		No performing arts training			
	*M* (*SD*)	percentile	*M* (*SD*)	Percentile	*t*(72)	*p*
General sophistication	97.89 (10.28)	76	71.54 (17.33)	32	7.95	<0.001
Active engagement	54.81 (5.85)	90	35.51 (8.30)	28	6.17	<0.001
Perceptual abilities	54.92 (4.68)	69	44.86 (7.84)	23	6.70	<0.001
Musical training	37.38 (4.67)	78	22.30 (8.87)	36	9.15	<0.001
Singing abilities	36.08 (4.14)	66	32.41 (4.62)	48	3.60	<0.001
Emotions	37.54 (7.43)	68	28.41 (7.24)	8	5.36	<0.001

### 3.2. Connectivity associated with musical sophistication

Table 3 reports the results of our analyses pertaining to Gold-MSI measures as the predictor variable on seed-to-voxel connectivity, organized by seed. Overall, we measured connectivity of 10 seeds associated with musical sophistication, spanning each of our Gold-MSI measures.

**Table 3:** Effect of Gold-MSI scales on resting state functional connectivity seed-to-voxel relationships across all participants.

Seed region	Gold-MSI measure	Cluster location	MNI coordinates	Cluster size	Relationship	*p*-FWE corrected
Sensory						
L Superior temporal gyrus (anterior)	Perception	L Frontal pole	−38 46 22	137	Negative	0.032
R Superior temporal gyrus (posterior)	Active	L Superior frontal gyrus	−20 34 46	130	Negative	0.037
		R Frontal pole	10 60 4	1,132	Negative	<0.001
		R Middle temporal gyrus (posterior)	40 18 –42	457	Negative	<0.001
Motor						
L Precentral Gyrus	Emotion	L Lateral occipital cortex (superior)	−24 −68 46	133	Negative	0.030
		R Postcentral gyrus	14–44 58	124	Negative	0.041
		R Precentral gyrus	8 –20 62	136	Negative	0.027
	General	L precentral gyrus (midline)	−2 −24 74	293	Negative	<0.001
	Training	R Lateral occipital cortex (inferior)	2 –32 56	261	Negative	<0.001
		R Lateral occipital cortex (inferior)	52 –72 12	197	Negative	0.004
R Precentral gyrus	Emotion	L Lateral occipital cortex (superior)	−26 −66 40	187	Negative	0.006
		L Postcentral gyrus*	8 –20 62	312	Negative	<0.001
		R Postcentral gyrus	14 –42 58	124	Negative	0.041
	Training	L Precentral gyrus (midline)	−4 −18 72	140	Negative	0.025
L Cerebellum	Emotion	R Insula	38 –6 2	134	Negative	0.013
Interoceptive						
L Insula	Emotion	L Parietal operculum	−54 −24 16	176	Negative	0.005
		R Frontal operculum	46 20 0	122	Negative	0.036
L Putamen	General	L Lingual gyrus	−4 −74 8	338	Positive	<0.001
		R Lingual gyrus	16 –54 −4	209	Positive	0.002
	Active	R Caudate	20 18 10	163	Negative	0.008
	Perception	L Lingual gyrus	−4 −74 8	138	Positive	0.020
		R Lingual gyrus	14 –54 −6	215	Positive	0.002
	Singing	L Lingual gyrus	−4 −80 −6	124	Positive	0.033
	Training	R Lingual gyrus	14 –54 −6	160	Positive	0.010
R Putamen	General	L Lingual gyrus	−4 −74 8	134	Positive	0.022
	Singing	L Frontal pole	−42 42 22	126	Negative	0.029
		L Lingual gyrus	−12 −72 −2	239	Positive	<0.001
L Thalamus	General	L Frontal pole	−38 50 24	142	Negative	0.024

*While the peak voxel of this cluster was localized in the right hemisphere, the majority of this cluster was localized over L Postcentral gyrus.

**Figure 2:**
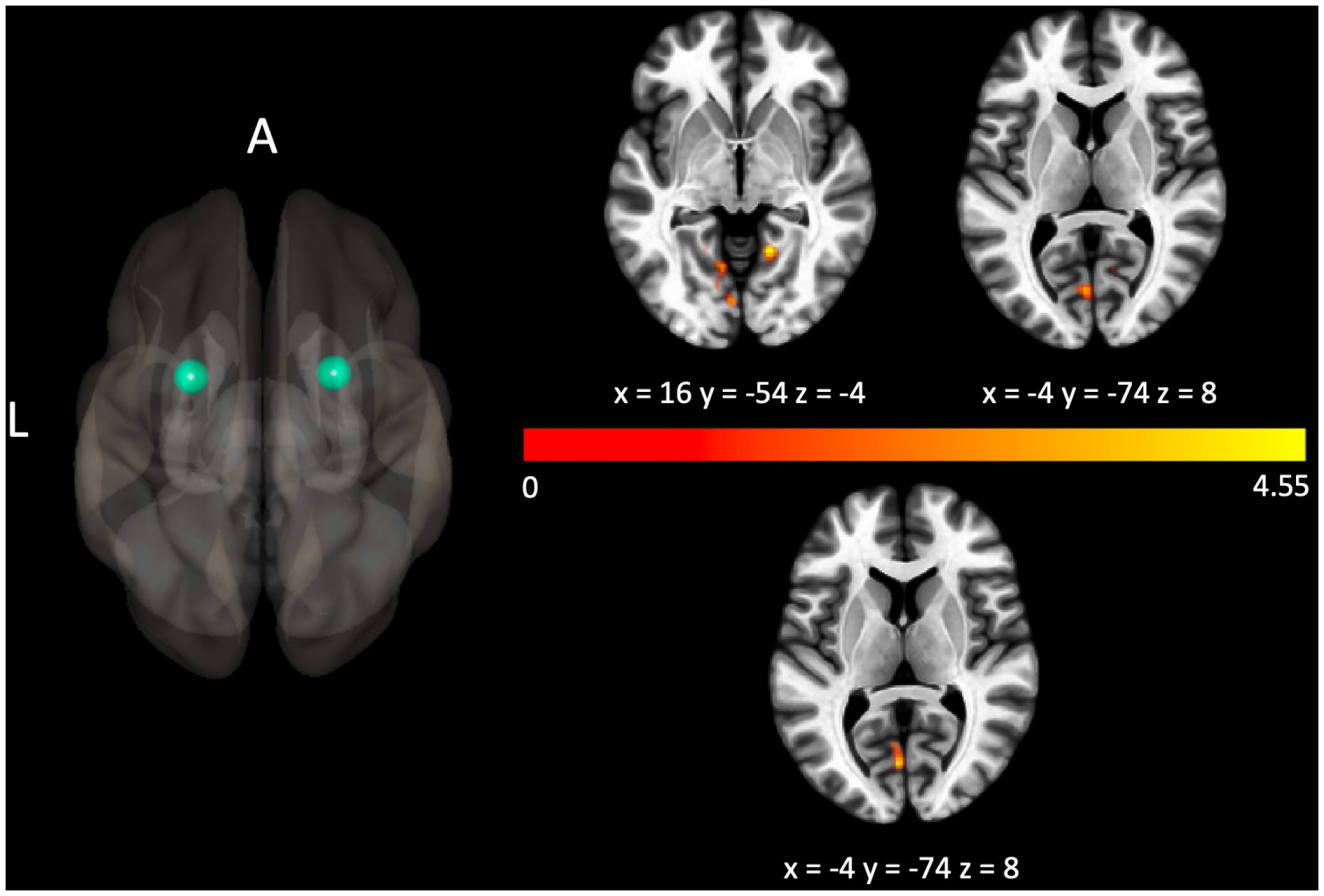
Connectivity between the seed localized to the left putamen and bilateral lingual gyri (above the colorbar), and the seed localized to the right putamen and the left lingual gyrus (below the colorbar) were positively associated with the Gold-MSI measure assessing general musical sophistication. For illustrative purposes, the seed regions are depicted as green spheres and the *t*-statistic of voxels in the associated clusters is indicated through warm colors to reflect the positive association.

The Gold-MSI measure assessing general musical sophistication was shown to be associated with connectivity related to four seeds. In particular, scores were negatively correlated with decreased connectivity between the left precentral gyrus and bilateral precentral gyrus, *p*_FWE_ < 0.001. Scores were positively correlated with connectivity related to bilateral putamen (with clusters localized over lingual areas, *p*_FWE_s < 0.05, shown in Figure 2), and the left thalamus (with a cluster over the left frontal pole, *p*_FWE_ = 0.024).

**Figure 3:**
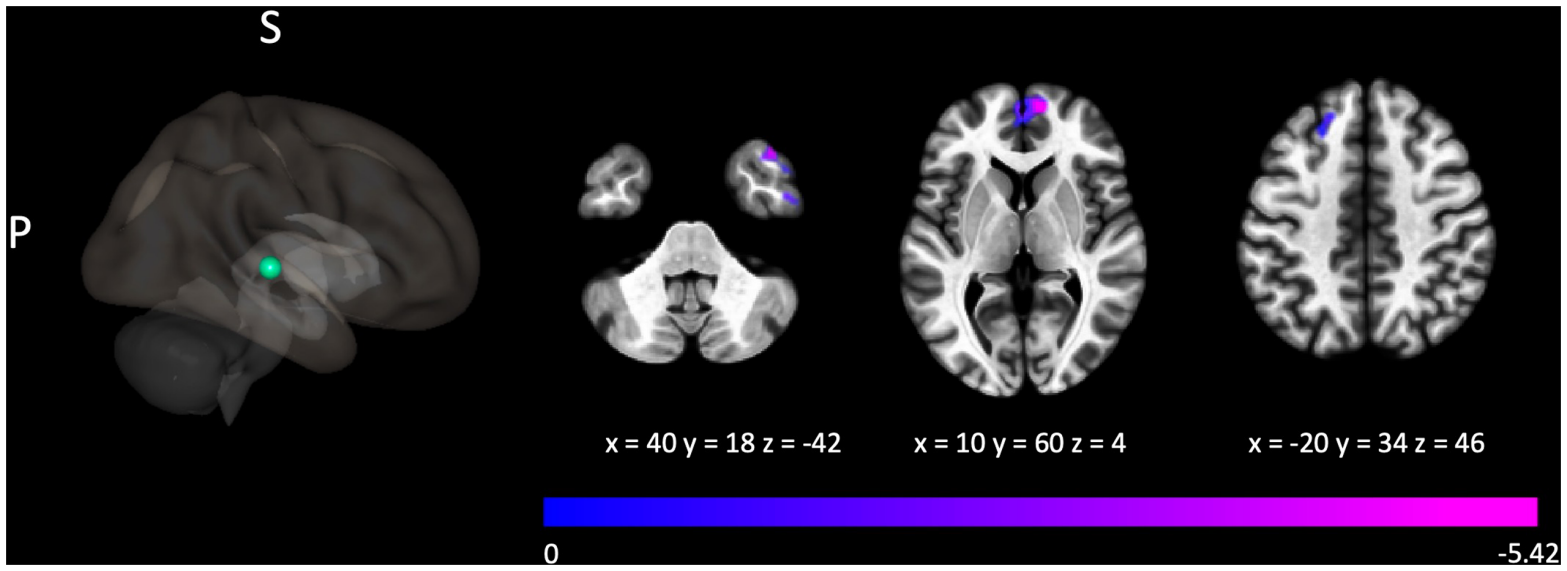
Connectivity between the seed localized to the right posterior superior temporal gyrus and clusters localized over the left superior frontal gyrus, right frontal pole, and right middle temporal gyrus was negatively associated with the Gold-MSI measure assessing active engagement. For illustrative purposes, the seed region is depicted as a green sphere and the *t*-statistic of voxels in the associated clusters is indicated through cool colors to reflect the negative association.

The Gold-MSI measure assessing active engagement was shown to be associated with connectivity related to the superior temporal gyrus, with clusters localized over the left superior frontal gyrus, *p*_FWE_ = 0.037, right frontal pole, *p*_FWE_ < 0.001, and right middle temporal gyrus, *p*_FWE_ < 0.001, shown in Figure 3. This Gold-MSI measure was shown to be associated also with connectivity related to the left putamen with a cluster over the right caudate, *p*_FWE_ = 0.008.

The Gold-MSI measure assessing perceptual ability was shown to be associated with connectivity related to the left superior temporal gyrus (with a cluster over the left frontal pole, *p*_FWE_ = 0.032) and left putamen (with clusters localized over bilateral lingual gyri, *p*_FWE_s < 0.05).

The Gold-MSI measure assessing musical training was shown to be associated with connectivity related to bilateral precentral gyri (with clusters localized over right lateral occipital cortex, *p*_FWE_s < 0.05, and left precentral gyrus although spanning the midline, *p*_FWE_ = 0.025). This measure was also associated with connectivity between the left putamen and right lingual gyrus, *p*_FWE_ = 0.010.

The Gold-MSI measure assessing singing abilities was shown to be associated with connectivity related to the bilateral putamen, between clusters localized over the left frontal pole, *p*_FWE_ = 0.029 and left lingual gyrus, *p*_FWE_s < 0.05.

**Figure 4:**
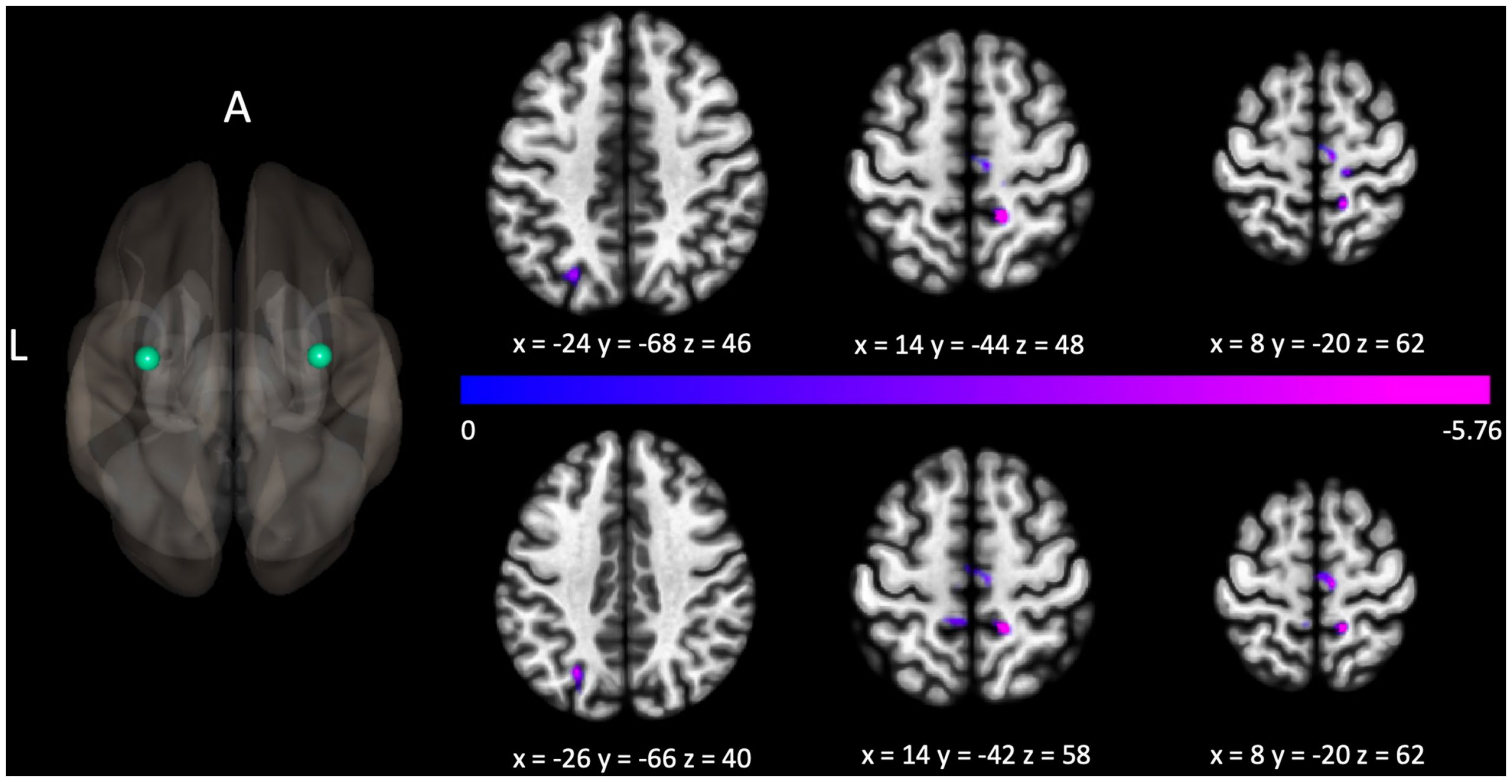
Connectivity between the seed localized to the left precentral gyrus and clusters localized over the left lateral occipital cortex, right post central gyrus, and right precentral gyrus (above the colorbar), and right precentral gyrus and clusters localized over the left lateral occipital cortex and bilateral post central gyri (below the colorbar), were negatively associated with the Gold-MSI measure assessing emotion. For illustrative purposes, the seed regions are depicted as green spheres and the *t*-statistic of voxels in the associated clusters is indicated through cool colors to reflect the negative association.

The Gold-MSI measure assessing emotions was shown to be associated with connectivity with connectivity related to bilateral precentral gyri (with clusters localized over left lateral occipital cortex, *p*_FWE_s < 0.05, right precentral gyrus, *p*_FWE_ = 0.027, and bilateral postcentral gyri, *p*_FWE_s < 0.05, shown in Figure 4). This measure was also associated with connectivity between the left cerebellum and a cluster localized over the left insula, *p*_FWE_ = 0.013, and between the left insula and clusters localized over left parietal operculum, *p*_FWE_ = 0.005, and right frontal operculum, *p*_FWE_ = 0.036.

### 3.3. Connectivity associated with performing arts training

Results of our analyses pertaining to between-group analyses are reported in Table 4. Between group analyses revealed that performance arts training modulated connectivity related to three seeds: left precentral gyrus, and bilateral posterior superior temporal gyrus. In particular, connectivity was decreased between the left posterior superior temporal gyrus and bilateral precentral gyri, *p*_FWE_ < 0.001, between the right posterior superior temporal gyrus and regions encompassing the right middle temporal gyrus, *p*_FWE_ = 0.010, and right frontal pole, *p*_FWE_ = 0.021, and between the left precentral gyrus and bilateral precentral gyri, *p*_FWE_ = 0.004, for participants who received performing arts training.

**Table 4:** Resting state functional connectivity seed-to-voxel relationships resulting from group-level comparisons.

	Seed	Cluster location	MNI coordinates	Cluster size	*p*-FWE corrected
Effect of performing arts training (formal vs. no formal training)					
Sensory					
	L Superior temporal gyrus (posterior)	R Precentral gyrus (midline)	6 –20 64	296	<0.001
	R Superior temporal gyrus (posterior)	R Frontal pole	8 64 –2	148	0.021
		R Middle temporal gyrus	60 2 –30	173	0.010
Motor					
	L Precentral gyrus	L Precentral gyrus (midline)	−8 −26 58	196	0.004
*Post hoc* analyses related to group comparisons with Gold-MSI scales included as covariate					
Gold-MSI Active^†^	R Superior temporal gyrus (posterior)	–	–	–	–
Gold-MSI General*	L Precentral gyrus	–	–	–	–

All contrasts were performed as two-sided tests, in the direction of formal performing arts training < No formal training. ^†^See the sensory section of Table 3. *See the motor section of Table 3.

Connectivity differences between performing arts training groups of the right posterior superior temporal gyrus and the left precentral gyrus thus resemble connectivity of these areas which relate to interindividual differences in the Gold-MSI scales measuring active engagement and general sophistication, respectively. Thus, we included Gold-MSI scores as covariates in *post hoc* analyses of the group differences. These analyses revealed that differences between groups were independent of differences in musical sophistication.

## 4. Discussion

Our results show that the resting-state connectivity of sensory, motor, and interoceptive brain areas, as identified in recent meta-analytic work (Pando-Naude et al., 2021; Criscuolo et al., 2022), relates to interindividual levels of musical sophistication. Overall, the uncovered co-activation patterns suggest that lower levels of musical sophistication are associated with greater connectivity of sensory and motor areas specifically. Similarly, our results also show greater connectivity of sensory and motor areas for participants who were not receiving performing arts training compared to their peers who were.

### 4.1. Discriminant validity of the Gold-MSI

Given the large number of participants who were enrolled in music classes at the time of the study, it is perhaps surprising that the connectivity differences between the groups of participants were most similar to those related to interindividual differences of general sophistication and active engagement rather than the music training aspect of the Gold-MSI. *Post hoc* analyses of the group differences which included the Gold-MSI scores as covariates showed that between-group differences were not explained by differences in musical sophistication in the general public and thus point toward performing arts-training specific effects.

Current training in the performing arts and musical sophistication may thus be associated with greater efficiency of the superior temporal gyri and the precentral gyri (see Tables 3, 4), while music training as an aspect of musical sophistication, which is primarily based on assessment of past training, may be more readily associated with connectivity between the precentral gyri and the right lateral occipital cortex, as well as the left putamen and the right lingual gyrus (see also below for a discussion on the relationship between expertise and the lingual gyri).

Closer inspection of our results further reveals that connectivity of sensory areas is associated with particular subscales of the Gold-MSI, namely those measuring active engagement and perceptual ability. In contrast, connectivity of motor areas is associated with the subscales of the Gold-MSI assessing musical training, emotions, and general sophistication. These results support the discriminant validity of the Gold-MSI subscales.

### 4.2. Mechanisms of musical sophistication

Further, we suggest potential mechanisms through which these aspects of musical sophistication may arise. As expected, music perceptual ability correlated with connectivity of the auditory cortex. Specifically, music perceptual ability correlated with connectivity patterns of the left anterior superior temporal gyrus. This result may shed further light on the specialization of the anterior superior temporal gyrus for processing complex musical sounds (Foo et al., 2016).

Connectivity of the right posterior superior temporal gyrus in contrast correlated with scores on the active engagement subscale, corroborating findings on its relevance for a number of music processing functions, particularly in musicians (Suarez et al., 2010; Angulo-Perkins et al., 2014). It should be noted that the performing arts training group scored particularly high on this subscale (see Table 2), and indeed, that the group comparison suggested that performing arts training further modulated connectivity of this brain area (see Table 4). Our results thus suggest that active engagement is modulated through connectivity in sensory areas (see Figure 3).

As hypothesized, connectivity of the motor cortex covaried with individual differences in music training. Specifically, connectivity between the left precentral gyrus with the right inferior lateral occipital cortex, and connectivity between the right precentral gyrus and the contralateral precentral gyrus correlated with scores on the training subscale. Our results thus add to the body of literature demonstrating the effects of music training on sensorimotor areas (e.g., Kleber et al., 2016, 2017).

Additionally, our results suggest that the emotional engagement with music is modulated through connectivity in motor areas (see Figure 4). Connectivity of the left and right precentral gyrus with clusters located right precentral, bilateral postcentral gyri, as well as the left superior lateral occipital cortex, and connectivity between the left cerebellum and right insula correlated with scores on the emotions subscale. Our results thus support other research showing the involvement of motor areas in the processing of emotions in music (Pereira et al., 2011; Putkinen et al., 2021), in particular, the involvement of the cerebellum in the processing of emotions in auditory input (Hopyan et al., 2010; Ceravolo et al., 2021). Further, they suggest that there are links between motor areas and aesthetic experiences in general (Kirsch et al., 2016).

### 4.3. Individual differences in musical sophistication

Individual differences in all subscales of the Gold-MSI were associated with connectivity of interoceptive and integrative areas, including the left insula, bilateral putamen, and thalamus (see Figure 2). Each of these areas have been implicated in studies on internal generation of musical structure or music imagery: For example, the putamen is thought to play a role in the internal generation of musical beat (Grahn and Rowe, 2009), while the thalamus and the basal ganglia are implicated in studies of musical imagery (Frisch et al., 2003; Kung et al., 2013; Pando-Naude et al., 2021).

Insula activity is known to be modulated by music and performing arts training (Luo et al., 2014; Kleber et al., 2017; Zamorano et al., 2017; Gujing et al., 2019; Ai et al., 2022). Our results add to this previous literature and support the idea that the insula and operculum may integrate mental imagery (Herholz et al., 2012) with interoceptive and emotional information during music performance (Tanaka and Kirino, 2016b), given the insula’s suspected role in linking information from diverse functional systems in the brain (Kurth et al., 2010).

Our results further suggest that singing abilities, as conceptualized for the Gold-MSI, are related to connectivity of the bilateral putamen. The bilateral putamen have been implicated in a number of studies on the processing of vocal auditory stimuli (Hopyan et al., 2010; Ceravolo et al., 2021; Thomasson et al., 2022). Thus, future research on the modulation of brain activity through singing training may consider including these areas as ROIs.

Overall, our results then suggest that the ability to internally represent musical structure or the ability to imagine music may determine individual levels of musical sophistication. However, the Gold-MSI does not directly characterize one’s use of music imagery. Further work will be necessary to explore how mental imagery modulates the effects of performing arts training or vice versa as well as the structural underpinnings of these patterns of connectivity.

### 4.4. Expertise and the lingual gyri

Our results further suggest that it may be the ability to internally represent musical sequences that determines individual levels of musical sophistication. Here, the connectivity of the putamen to the bilateral lingual gyri is positively associated with different aspects of musical sophistication. This increased striatal functional connectivity with lingual gyri has been previously observed in musicians compared to non-musicians and is thought to relate to individual expertise in reading musical notation given the lingual gyrus’ role in higher order pattern recognition (van Vugt et al., 2021).

Expertise is also reflected by activity of the lingual gyri in music (Neumann et al., 2016) and sport (Guo et al., 2017; Kraeutner et al., 2022). For instance, improved motor performance on a sport-related task (e.g., dart throwing, table tennis) is linked to decreased activity of the lingual gyri during both mental rehearsal of the same task (Kraeutner et al., 2022) and perceptual priming using stimuli related to the same task (Guo et al., 2017).

However, it should be noted that the connectivity of some of the included seeds was not associated with musical sophistication, including Heschl’s gyri. This may come as a surprise given that structural and functional differences of Heschl’s gyri between participants with and without music training are well-documented (Schneider et al., 2002; Gaser and Schlaug, 2003; Hyde et al., 2009; Kleber et al., 2016; Jiang et al., 2023). These results thus help further delineate between music training and musical sophistication which are overlapping but not identical concepts (Müllensiefen et al., 2014). Future work should also investigate the relationship between individual differences in musical sophistication and variation in white matter tracts supporting the function of Heschl’s gyri.

A major limitation of our study is the possibility of increased Type 1 error given the number of seeds included in our analyses and large numbers of statistical comparisons performed even though all of our results were corrected for multiple comparisons within each seed. It is also important to consider the different subtypes of performing arts training. As we recruited generally from performing arts classes future work is needed to investigate specific effects of music training or particular effects of acting training. However, we believe that our work is impactful given that it includes a relatively large sample size, with multiple control conditions. Thus, it may serve to help generate new hypotheses. It also extends past work conducted using functional connectivity measures to further understanding of how performing arts training impacts the brain.

## 5. Conclusion

In conclusion, we assessed (1) correlations of resting-state functional brain connectivity and musical sophistication, and (2) the effects of performing arts training. We found that overall, the connectivity of sensory and motor areas may be associated with individual levels of specific aspects of musical sophistication. Further, we discovered that individual levels of musical sophistication also correlate with differences in the connectivity of interoceptive and integrative areas. Lastly, performing arts training may be associated with connectivity of sensory and motor areas over and above individual differences in this connectivity associated with musical sophistication. In the future, analyses may also consider structural differences of these areas.

## Data availability statement

The raw data supporting the conclusions of this article will be made available by the authors, without undue reservation.

## Ethics statement

The studies involving humans were approved by the University of British Columbia Office of Research Ethics. The studies were conducted in accordance with the local legislation and institutional requirements. The participants provided their written informed consent to participate in this study.

## Author contributions

A-XC, NMY, NH, JW, and LB conceptualized the study. A-XC and NMY collected the data. A-XC and SK carried out the data analysis and wrote the first draft. All authors contributed to the manuscript.
